# An Analysis of the Preoperative Factors, Spinopelvic Mobility and Sagittal Spinal Alignment in Pre-THA Patients

**DOI:** 10.3390/jcm12175594

**Published:** 2023-08-27

**Authors:** Mariusz Łaziński, Włodzimierz Niemyjski, Michał Niemyjski, Marek Synder, Marek Drobniewski, Łukasz Olewnik, Andrzej Borowski

**Affiliations:** 1 Oddział Chirurgii Urazowo-Ortopedycznej, Szpital Wojewódzki im. Jana Pawła II w Bełchatowie, 97-400 Bełchatów, Poland; lazinski.mariusz@gmail.com (M.Ł.); wni@onet.pl (W.N.); mni@vp.pl (M.N.); 2 Orthopaedics and Paediatrics Orthopaedics Clinic, Medical University of Lodz, 90-151 Lodz, Poland; msynder@pro.onet.pl (M.S.); marek.drobniewski@umed.lodz.pl (M.D.); 3 Department of Anatomical Dissection and Donation, Medical University of Lodz, 90-151 Lodz, Poland; lukasz.olewnik@umed.lodz.pl

**Keywords:** spinopelvic mobility, sagittal spinal alignment, total hip arthroplasty, hip spine classification, dislocation

## Abstract

Hip arthroplasty is a very effective medical procedure. The optimal positioning of the components and the functioning of the endoprosthesis are influenced, among other things, by the mobility and balance of the spine. The aim of the study was to analyze the factors influencing the mobility of the lumbar–pelvic–iliac complex (spinopelvic mobility) together with the assessment of sagittal spinal alignment in patients prior to THA (total hip arthroplasty). Patients who underwent hip replacement surgery due to advanced osteoarthritis of the hip were enrolled in the study (n = 103). The sociodemographic characteristics, BMI, radiological advancement of the degenerative disease, quality of life, and range of joint mobility were completed using a proprietary questionnaire, the EQ-5D-5L questionnaire, and a clinical examination. X-ray images were analyzed: AP of the pelvis standing up, lateral of the spine standing and sitting. Key parameters were measured as ∆SS—change in sacrum angle value when changing from standing to sitting position and pelvic incidence (PI)—lumbar lordosis (LL) mismatch—sagittal lumbar pelvic balance measured in standing position. The patients were assigned to the appropriate groups according to the Hip-Spine Classification: normal group: 1A (n = 65; 63.1%), abnormal groups: 1B (n = 17; 16.5%), 2A (n = 16; 15.5%), 2B (n = 5; 4.9%). A correlation was shown between the abnormal groups and the individual components of PROMs in the scope of the self-service and normal activities categories (EQ-5D and EQ-VAS). However, the strength of the relationship turned out to be moderate, and the remaining components of the survey were statistically insignificant. The remaining factors analyzed, i.e., age, BMI, the range of hip motion, the presence of contracture in the joint in a clinical examination, and the radiological advancement of osteoarthritis on the Tonnis scale, do not predict abnormal relationships between the spine and the pelvis in our patients waiting for THA. Therefore, further investigations are needed to evaluate the correlation between preoperative factors and the lumbar–pelvic–iliac complex in patients prior to planned hip arthroplasty.

## 1. Introduction

Osteoarthritis is one of the most common causes of disability in the world, especially in people over 50 [1]. The advanced condition leads to persistent pain, reduced physical capacity, and difficulty in carrying out activities of daily living [2]. The surgical method of treatment is hip arthroplasty, which is in fact a very effective medical procedure; this procedure is becoming increasingly common due to the aging of the population and increased life expectancy [3]. Complications include pulmonary embolism (VTE), peri-prosthetic infection (PJI), peri-prosthetic fracture, endoprosthesis instability, and limb unevenness [4]. In some cases, the endoprosthesis may also be dislocated [5]. They are the leading cause of failure in arthroplasty and reoperation [6,7]. 

Large multicenter studies estimate total postoperative hip instability to range from 2–4% of all procedures [8,9,10,11]. In order to correctly position the prosthetic components, the type of surgical access, the failure of the abductor muscles, and the choice of implants and articulations based on preoperative planning appear to be reasonable courses to be considered [9]. 

An important etiological factor of THA (total hip arthroplasty) instability is also a malfunction of the pelvic–spine complex [10,11]. In recent years, much attention has been paid to the interaction between the hip, pelvis, and spine [7,12]. The mobility of the lumbar–pelvic–iliac complex depends on the change in body posture from standing to sitting. Lumbar lordosis decreases (ΔLL), the hip joint is flexed (ΔPFA—proximal femoral angle), the pelvis tilts backwards (Δ APPt—anterior pelvic plane tilt), and the angle of the sacrum decreases (∆SS—sacral slope). Incorrect mobility of the complex is commonly classified as stiffness (∆SS < 10°) or hypermobility (∆SS > 30°) when changing position from standing to sitting [13]. Patients with insufficient spine mobility are at increased risk of endoprosthesis dislocation and a poorer surgical outcome [14].

Sagittal balance is assessed based on the difference between pelvic incidence (PI) and lumbar lordosis (LL) in a standing position. A PI-LL (pelvic incidence-lumbar lordosis) mismatch > 10° on the lateral X-ray image indicates a flat deformity of the back. Awareness of this abnormality could help surgeons performing hip arthroplasty decrease the risk of complications in this group of patients [15].

The aim of the study was to identify possible predictive factors for abnormal mobility and balance of the lumbar–pelvic–iliac complex in patients with advanced osteoarthritis of the hip before arthroplasty without the need to take additional X-rays in the lateral projection. The following variables were analyzed: (1) age; (2) BMI; (3) PROMs collected using the EQ-5D-5L and EQ-VAS survey; (4) ROM of the hip and the presence of contracture in the joint in a clinical examination; and (5) radiological advancement of degenerative disease on the Tonnis scale.

## 2. Materials and Methods

The prospective study involved 103 patients (men, n = 57; 55.3%, women, n = 46; 44.7%) with a diagnosis of osteoarthritis of the hip. Each patient received extensive information about the clinical trial and signed their written informed consent to take part. Patients undergoing partial hip arthroplasty or revision arthroplasty, diagnosed with a hip fracture, improperly taken X-rays, and underweight BMI < 18.5 kg/m^2^ or severely obese BMI > 40 kg/m^2^ were excluded. Sociodemographic characteristics, BMI, quality of life, and the range of joint mobility were completed using a proprietary questionnaire, the EQ-5D-5L + EQ-VAS questionnaire, and a clinical examination. The basic data characterizing the study group are presented in Table 1.

BMI was calculated according to the standard formula—body weight (kg)/height (m)^2^, then the patients were divided into three groups according to the WHO definition: Group 1: normal BMI ≥ 18.5–24.9 kg/m^2^ (n = 21; 20.6% ); Group 2: overweight ≥ 25.0–29.9 kg/m^2^ (39; 38.2%); Group 3: obese ≥ 30–39.9 kg/m^2^ (n = 42; 41.2%) [16].

The questionnaires were collected based on the Euro-Quality of Life Questionnaire EQ-5D ver. 5L [17,18], which allows for self-assessment of patient health. It considers five dimensions of quality of life assessed on a scale from 1 (least problems/ailments) to 5 (greatest problems/ailments) reflecting their current state: moving, self-service, ability to perform ordinary activities, feeling pain/discomfort, feeling anxious/depressed. The respondents also completed the Visual Analogue Scale (EQ-VAS, EuroQol Visual Analogue Scale, EuroQol Research Foundation: Rotterdam The Netherlands). This scale makes it possible to obtain information about the health condition at a given moment and ranges from 0 (worst) to 100 (best) (20). The official consent of the EuroQol Group was obtained to conduct the study using the EQ-5D-5L questionnaire in Polish.

The range of passive hip mobility, such as flexion, abduction, adduction, internal rotation, external rotation, and the deficit of passive extension, was measured in a clinical study using a standard goniometer. Classical goniometer measurement is believed to be a reliable and appropriate method of determining hip ROM [19].

The severity of the osteoarthritis of the examined hip joint was expressed according to the Tonnis classification based on a pelvic X-ray covering both hip joints, taken in the standing AP projection. The Tonnis classification consists of three progressive degrees of hip degeneration: Grade 0 indicates no degeneration; Grade 1 is characterized by slight joint space narrowing, slight joint edge tightening, and slight sclerotization; Grade 2 by the presence of small subchondral cysts, further joint space narrowing and moderate loss of sphericity of the femoral head; and Grade 3 by large subchondral cysts, severe joint space narrowing, severe femoral head deformity, and aseptic necrosis [20]. The results are presented in Table 2.

The radiological analysis of the mobility of the lumbar–pelvic–iliac complex and the balance in the sagittal plane was based on the following X-ray images, taken before hip arthroplasty:

1. Lateral X-ray of the spine and pelvis from L1 to the proximal end of the femur while standing.
2. Lateral X-ray of the spine and pelvis from L1 to the proximal end of the femur in a sitting position (relaxed position in a chair adjusted to patient height, knee and hip joints bent to 90°, thighs parallel to the floor [21]. The relaxed seated position more adequately classifies the spinopelvic mobility compared to the straight seated position when obtaining preoperative spinopelvic radiographs [22].

**Figure 1:**
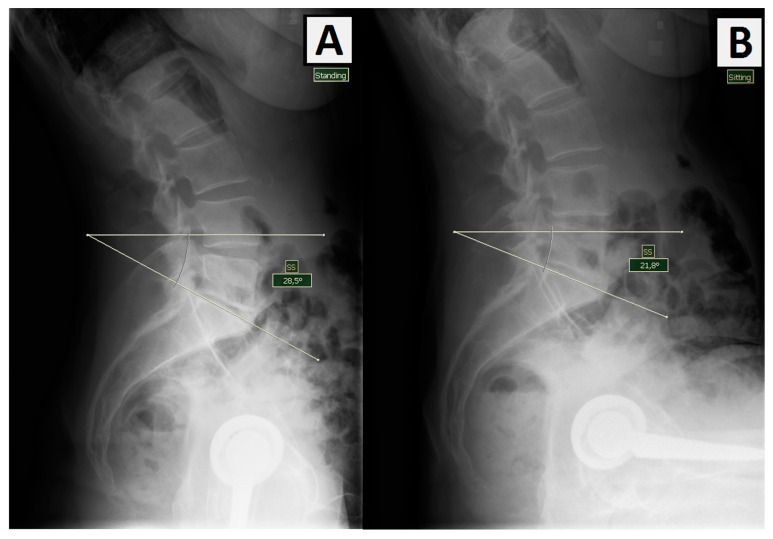
Measurement of the angle of inclination of the sacrum (SS) in the (**A**) standing position and (**B**) sitting position where ∆SS is 6, 7°.

**Figure 2:**
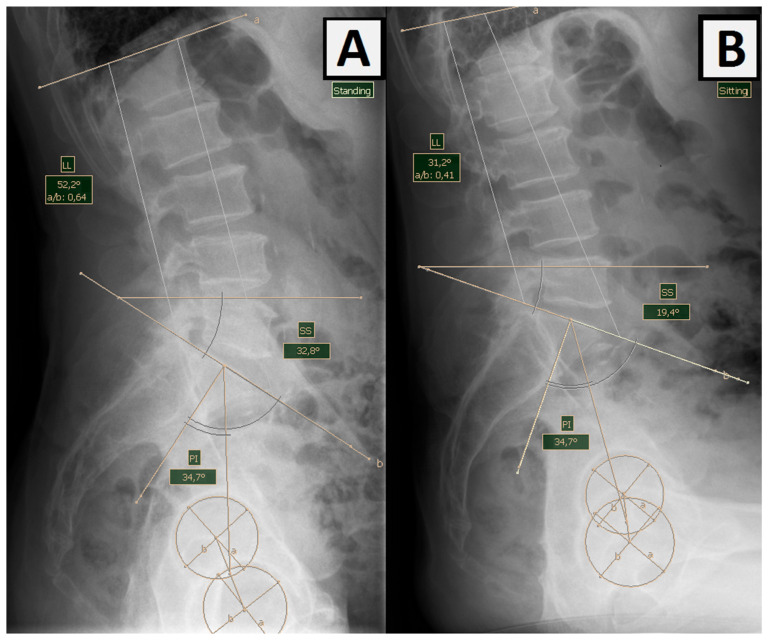
Measurement of lumbar lordosis (LL) and pelvic incidence (PI) angles in (**A**) standing and (**B**) sitting positions. The ∆LL of this patient is 21°, while the PI remains unchanged 34,7°. PI-LL measured in an upright position is −17.5°.

Measured parameters:

1. ∆ sacral slope (tilt) (∆SS)—change in the angle of the sacrum while changing the position from standing to sitting (the angle between the horizontal line and the tangent to the border plate of the sacrum) (Figure 1). This measurement helps identify patients with spinal stiffness or hypermobility [23].

2. Pelvic incidence (PI)—lumbar lordosis (LL) mismatch (PI-LL)—sagittal lumbar pelvic balance measured in a standing position using PI (the angle between the straight line connecting the center of the femoral heads with the center of the S1 border plate and the line perpendicular to the base of the sacrum) and LL (lumbar lordosis curve angle) (Figure 2) [24]. Pelvic incidence is a morphological parameter that remains constant despite the movement of the spine and pelvis throughout adulthood and does not change as a result of degenerative diseases of the spine. Pelvic incidence-lumbar lordosis mismatch (PI-LL mismatch) is a parameter often used by spine surgeons to define sagittal deformities. For arthroplasty surgeons, it is an important measure to help identify patients with flat deformities of the back resulting from the abolition of lordosis and a posterior tilt of the pelvis in a standing position (Figure 2) [25].

## 3. Analysis

The mobility of the lumbar–pelvic–iliac complex when changing from standing to sitting was assessed as follows: ∆SS < 10°—stiffness, ∆SS > 10°—preserved mobility. The balance of the lumbar–pelvic–iliac complex in the sagittal plane in the standing position was interpreted as follows: PI-LL > 10°—mismatch, imbalance, and planar deformity of the spine; PI-LL < 10°—balance is preserved.

Patients were assigned to four groups according to the Hip-Spine Classification:

1A. Correct balance (PI-LL) < 10° and normal mobility (∆SS > 10°)

1B. Normal balance (PI-LL) < 10° and abnormal mobility (∆SS < 10°)

2A. Incorrect balance (PI-LL) > 10° and normal mobility (∆SS > 10°)

2B. Abnormal balance (PI-LL) > 10° and abnormal mobility (∆SS < 10).

The results were analyzed based on Spearman’s monotonic correlation coefficient, the Shapiro–Wilk test, and the 2-association test. The level of significance was α = 0.05.

## 4. Results

The obtained values of the changes in the angle of inclination of the sacrum while changing the position from standing to sitting and the difference in PI-LL parameters in the standing position are presented in Table 3. 

Patients were assigned to the appropriate groups according to the Hip-Spine Classification: 1A (n = 65; 63.1%), 1B (n = 17; 16.5%), 2A (n = 16; 15.5%), 2B (n = 5; 4.9%), which are presented in Table 4 in comparison to similar studies.

Groups 1A (n = 65; 63.1%) and abnormal groups 1B, 2A, 2B (n = 38; 36.9%) were compared using the χ association test and the monotonic rho-Spearman correlation coefficient with regard to the following variables: (1) age (i.e., <65 years or >65 years of age); (2) BMI; (3) PROMs collected using the EQ-5D-5L and EQ-VAS survey; (4) the range of hip mobility and the presence of contracture in the joint based on clinical examination; (5) radiological advancement of degenerative disease (Tonnis scale) and disorders in the spine. 

No statistically significant correlation was found between the studied variables in terms of age, body weight (BMI), hip extension deficit, or radiological advancement of degenerative disease. The results of the χ test are presented in Table 5.

In the case of the passive range of hip joint motion, the results also do not indicate any significant dependencies. The results of the Spearman rho correlation analysis are presented in Table 6.

A statistically significant correlation was found for the self-service, usual activities and health today (0–100) (Eq-5D-5L and EQ-VAS) and Hip-Spine Classification group, i.e., correct (1A) or incorrect (1B, 2A, 2B) (Table 7).

**Figure 3:**
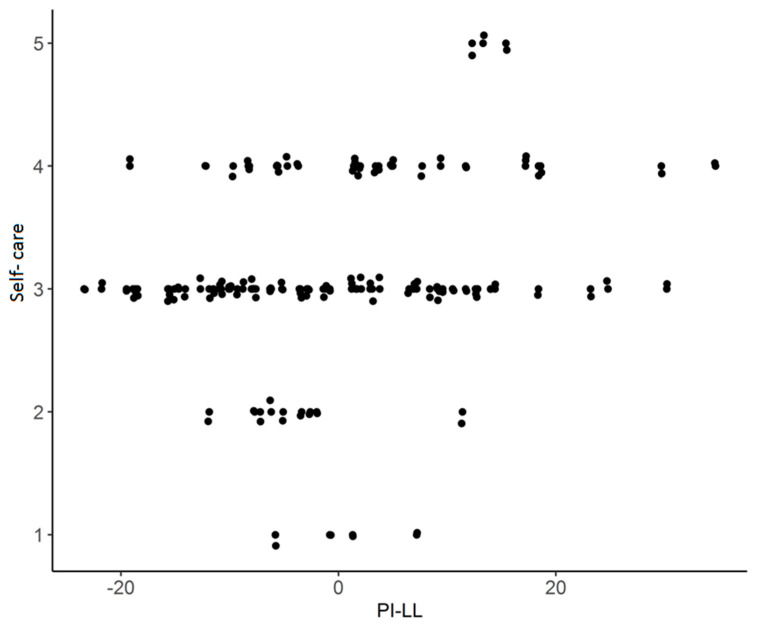
The relationship between “PI-LL” and “self-care”.

The strength of the correlation in all variables turned out to be moderate, and the direction of dependence in terms of self-service and normal activities turned out to be negative—higher values on the scale 1–5 occurred in patients in the abnormal group (1B, 2A, 2B), while on a 0–100 scale, the direction of correlation turned out to be positive, with higher values in the normal group (1A). The statistically significant components of the questionnaire depended on the values of the ∆SS and PI-LL angles. The results of the Spearman rho correlation analysis show the existence of a significant relationship between the variables: PI-LL—self-service, rho = 0.25 *, 95% CI [22], *p* = 0.010, n = 103—the strength of the correlation turned out to be moderate, and the direction of the correlation turned out to be positive: self-service increases significantly with the increase in the PI-LL (Figure 3).

## 5. Discussion

The conducted study holistically presents the radiological relationships between the spine and the pelvis in patients diagnosed with osteoarthritis of the hip joint. Unlike in other publications, patients with newly diagnosed spinal pathology or a history of disease have not been excluded, making the cohort more representative of patients reporting for surgery [27]. The scale of the problem is emphasized by the fact that abnormalities were detected in as many as 36.9% of patients. Some authors suggest that side X-rays should be taken standing and sitting in patients waiting for THA, which will optimize implantation of the prosthetic components to avoid impingement, reduce the risk of dislocation and wear, and improve postoperative outcomes [28]. However, routinely making additional projections in several positions increases the cost and significantly increases the radiation dose. Accordingly, our analysis includes a number of factors that would allow clinicians to predict abnormalities of the lumbar–pelvic–thoracic complex and avoid taking additional X-rays.

As in other studies, significant diversity was observed in the parameters describing the mobility of the spine [9]. The highest tilt of the sacrum back when changing from standing to sitting was 42.2°, and a few patients demonstrated the reverse tendency (forward leaning) by a max. 3.5°. The sagittal mismatch of the PI-LL in the standing position also showed high inter-individual variability, ranging from −23.5° to 34.7° with a mean value of 0.10° ± 12.038°, which is close to Haffer et al.’s outcome (3.1 ± 13.3) [29].

Based on ΔSS and PI-LL measurements, patients were divided according to the Hip Spine Classification: 1A (63.1%), 1B (16.5%), 2A (15.5%), and 2B (4.9%); similar results were achieved as in a previous multicenter study by Vigdorchik and coefficients: 1A (47.4%), 1B (11.1%), 2A (34.5%), and 2B (7%) [26]. Ohyama Y et al. presented results showing that there are significantly more patients in abnormal groups than in groups without pathology (Table 4) [22]. 

These findings suggest that abnormalities in the spine in patients undergoing THA are a common issue. Extended and modified preoperative planning based on ΔSS and PI-LL measurements seems particularly appropriate in these patients. According to some authors, additional lateral X-ray images should be taken, especially in patients with lumbosacral spondylosis, kyphotic standing, degenerative diseases in the spine, and contracture in the hip joint [13]. Since low PI, PI-LL mismatch, and low SS during sitting and standing may contribute to the risk of prosthetic impingement followed by dislocation, great care should be taken to prevent the loss of spinopelvic mobility after spinal fusion surgery.

Pre-operative identification of patients with spinal abnormalities would assist surgeons with pre-operative planning, implant selection, and intra-operative positioning. Many authors emphasize the importance of spine mobility and sagittal balance, but there are still no clear guidelines on how to screen patients with pathology of the lumbar–pelvic–iliac complex or which tools to use [16,25,30]. Currently, the only effective method is to take a series of additional lateral X-ray images in a standing and sitting view of all patients waiting for arthroplasty, which is associated with additional radiation exposure and has economic consequences [31]. The contribution to future studies and the importance of the study is to assess which patient-related factors are strongly related to spine abnormalities so hip surgeons can avoid performing total hip arthroplasty in these patients without taking additional lateral X-rays. 

However, the study has a few limitations. Radiological diagnostics were performed during hospitalization, and patients were classified into risk groups on the basis of preoperative photos. After the procedure, only X-rays of both AP hips were taken (without lateral images), which justifies the assumption that the parameters may change [5]. Studies show that the mobility of the spine and the balance in the sagittal plane in the standing and sitting positions change only to a small extent or not at all [30,32]. However, Yun et al. showed that although preoperative SS correlated strongly with postoperative SS in the supine and standing positions and change in SS was minimal by THA overall, there was high variability to be clinically relevant, especially in the sitting position and spinopelvic mobility, over ±7° changes [33].

Hence, preoperative measurements may, to some extent, predict the relationship between the spine and the pelvis after arthroplasty. In addition, the above two functional items were selected for evaluation as they have been the most extensively studied and discussed elsewhere. As a consequence, no other photos were taken, e.g., in deepened flexion while sitting or in hyperextension [34]. However, further testing in various static and dynamic positions is advisable. Another limitation was the possibility of taking X-ray images only of the lumbar spine and the proximal parts of the femurs, without the cervical and thoracic spine due to the lack of access to postural images. Thus, the potential dependencies of other parts of the spine in relation to mobility and sagittal fit could not be investigated [35]. Nevertheless, the projections made allowed assigning the patients to the appropriate groups according to the Hip-Spine Classification.

Although the individual components of the PROMs measured with the EQ-5D-5L and EQ-VAS were found to be statistically significant, the allocation of patients to groups with abnormalities in the spine and pelvis, the above-mentioned factors. Apart from hip and spine disease, there are many aspects that reduce the quality of life, and the assessment may change over time [36]. In addition, the studies were carried out during the hospitalization of patients with diagnosed advanced degenerative disease before arthroplasty, i.e., after unsuccessful, often many years of conservative treatment, and before giving a chance to reduce symptoms and improve the function of surgical treatment. The quality of life of patients in the study population was significantly reduced in all patients (mean EQ-VAS 50.53).

We currently have a short time (one year) to observe postoperative complications and confirm the relationship between potential dislocations and patient assignment to a group based on Hip-Spine Classification. We plan a long-term follow-up to verify the relationship between patients in abnormal groups and the instability of the prosthesis components.

## 6. Conclusions

The problem of abnormal relationships between the spine and the pelvis in patients waiting for THA was analyzed and determined, and the factors significantly influencing the reduction of postoperative outcomes were presented. Abnormalities of the lumbar–pelvic–iliac complex, indicated by Hip-Spine Classification and self-service and normal activities values (EQ-5D and EQ-VAS) indicate lower performance among patients in the abnormal groups (1B, 2A, 2B); however, the health today 0–100 score indicates better well-being in patients in the normal group (1A). However, the strength of the relationship turned out to be moderate, and the remaining components of the survey were statistically insignificant. The remaining factors analyzed, i.e., age, BMI, hip ROM, the presence of contracture in the joint in the clinical examination, and the radiological advancement of osteoarthritis according to the Tonnis scale, did not allow us to predict abnormal relationships between the spine and the pelvis in patients waiting for THA in our study. We thought that a minimum number of X-rays were needed to evaluate the lumbar–pelvic–iliac complex. However, none of our assessed factors can statistically rule out the presence of pathology in the spine. Therefore, more studies are needed to evaluate the correlation between preoperative factors and the lumbar–pelvic–iliac complex by taking lateral X-rays of the spine and pelvis while standing and sitting. However, we find the lateral view of this complex and ∆SS and PI-LL measurements useful as a good tool to predict the outcome of total hip replacement. This valuable information serves to focus the preoperative screening on the THA candidates with the highest risk for abnormal spinopelvic function and could help surgeons in implant positioning or implant selection (use of dual-mobility articulation).

Patient classification according to their spinopelvic mobility and sagittal spinal balance appears to be significant because not only ours but many other publications suggest that the problem is very frequent and severe. Since there is no research that can identify and statistically predict spinal pathology based on preoperative factors, it appears reasonable to further investigate this correlation in order to avoid taking additional X-rays. 

## Figures and Tables

**Table 1:** Basic descriptive statistics of the study group.

	*N*	*M*	*SD*	*Me*	Min.	Max.	Tendency	Kurt
Age	103	66.41	9.359	68.00	30.00	84.00	−0.99	1.50
BMI	103	28.83	4.142	28.41	17.87	38.09	0.02	−0.64
Mobility	103	3.41	0.747	3.00	2.00	5.00	−0.25	−0.51
Self-care	103	3.16	0.777	3.00	1.00	5.00	−0.39	1.03
Usual activities	103	3.50	0.803	4.00	1.00	5.00	−0.66	0.59
Pain/discomfort	103	4.09	0.864	4.00	1.00	5.00	−0.71	0.23
Anxiety/depression	103	3.20	0.662	3.00	1.00	4.00	−0.44	0.05
Health today 0–100	103	50.53	8.629	50.00	30.00	75.00	0.28	0.20
Flexion	103	88.11	13.138	90.00	30.00	120.00	−0.83	3.32
Extension deficiency	24	8.96	4.56	10.00	5.00	20.00	1.04	0.25
Abduction	103	21.46	10.610	20.00	0.00	45.00	0.19	−0.59
Adduction	103	11.07	6.703	10.00	0.00	30.00	0.53	0.39
Internal rotation	103	5.17	7.002	3.00	0.00	30.00	1.59	2.31
External rotation	103	13.79	11.493	10.00	0.00	45.00	0.90	0.26

N—number of valid observations, M—mean, SD—standard deviation, Me—median, Min—minimum value, Max—maximum value.

**Table 2:** Radiological advancement of the disease on the Tonnis scale.

	*N*	*n* (%)
Degree	103	
1		8 (7.8%)
2		36 (35.0%)
3		59 (57.3%)

**Table 3:** Values of the changes in the angle of inclination of the sacrum and PI-LL parameters.

	*N*	*M*	*SD*	*Me*	Min.	Max.	Tendency	Kurt
ΔSS	103	18.97	10.686	19.50	−3.50	42.20	0.06	−0.53
PI-LL	103	0.10	12.038	−1.20	−23.40	34.70	0.45	−0.11

N—number of valid observations, M—mean, SD—standard deviation, Me—median, min.—minimum value, max.—maximum value.

**Table 4:** Number and percentage of patients in each of the four groups according to the Hip-Spine Classification.

	Group	1A	1B	2A	2B
	Classification	PI-LL < 10° and ΔSS > 10°	PI-LL < 10° and ΔSS < 10°	PI-LL > 10° and ΔSS > 10°	PI-LL > 10° and ΔSS < 10°
Relaxed seated position	Patients, *n* (%)	65 (63.1)	17 (16.5)	16 (15.5)	5 (4.9)
Vigdorchik et al. [26]	Patients, *n* (%)	987 (47)	232 (11)	715 (34)	147 (7)
Ohyama Y et. al. [22]	Patients, *n* (%)	19 (25)	7 (9)	33 (44)	16 (21)

PI-LL, pelvic incidence minus lumber lordosis angle; ΔSS, change in sacral slope from the standing to the relaxed seated position.

**Table 5:** Correlation between the examined variables and the correct (1A) and incorrect (1B, 2A, 2B) group according to Hip-Spine Classification.

Variables	*n*	χ^2^	*df*	*p*	Cramer’s *V*
Age (≥65 y.o. i < 65 y.o.)	103	0.0	1	0.949	0.01
BMI (≥18.5–24.9, ≥25.0–29.9^2^, ≥30–39.9)	103	1.3	2	0.529	0.11
Tonnis scale (I, II, III)	103	0.9	2	0.644	0.09
Hip extension deficit	103	0.1	1	0.755	0.03

**Table 6:** The results of the analysis of the rho-Spearman correlation between the individual components of the Hip ROM in the normal (1A) and abnormal groups (1B, 2A, 2B) according to Hip-Spine Classification.

95% CI					
ROM	*n*	rho	Lower	Upper	*p*
Flexion	103	−0.03	−0.22	0.17	0.799
Adduction	103	0.14	−0.06	0.33	0.156
Internal rotation	103	0.02	−0.18	0.22	0.858
External rotation	103	0.02	−0.18	0.22	0.850
Abduction	103	0.06	−0.14	0.26	0.544

**Table 7:** The results of the analysis of the rho-Spearman correlation between the individual components of EQ-5D i EQ-VAS in the normal (1A) and abnormal groups (1B, 2A, 2B) according to Hip-Spine Classification.

95% CI						
EQ-5D i EQ-VAS	*n*	rho		Lower	Upper	*p*
Pain/discomfort	103	−0.01		−0.21	0.19	0.931
Anxiety/depression	103	−0.14		−0.33	0.06	0.157
Mobility	103	−0.17		−0.36	0.03	0.085
Self-care	103	−0.23	*	−0.41	−0.04	0.018
Usual activities	103	−0.23	*	−0.41	−0.04	0.018
Health today 0–100	103	0.23	*	0.03	0.41	0.022

* *p* < 0.05.

## Data Availability

All data supporting reported results are available on request.
